# Anti-Tumor Effects of Wee1 Kinase Inhibitor with Radiotherapy in Human Cervical Cancer

**DOI:** 10.1038/s41598-019-51959-3

**Published:** 2019-10-28

**Authors:** Yoo-Young Lee, Young-Jae Cho, Sung-won Shin, Changhoon Choi, Ji-Yoon Ryu, Hye-Kyung Jeon, Jung-Joo Choi, Jae Ryoung Hwang, Chel Hun Choi, Tae-Joong Kim, Byoung- Gie Kim, Duk-Soo Bae, Won Park, Jeong-Won Lee

**Affiliations:** 1 Department of Obstetrics and Gynecology, Samsung Medical Center, Sungkyunkwan University School of Medicine, Seoul, Korea; 2 Department of Radiation Oncology, Samsung Medical Center, Sungkyunkwan University School of Medicine, Seoul, Korea; 3 Samsung Biomedical Research Institute, Samsung Medical Center, Sungkyunkwan University School of Medicine, Seoul, Korea; 4 Institute for Refractory Cancer Research, Samsung Medical Center, Seoul, Korea; 5 Samsung Advanced Institute for Health Sciences & Technology, Sungkyunkwan University School of Medicine, Seoul, Korea

**Keywords:** Cancer models, Targeted therapies, Cervical cancer

## Abstract

Although the concurrent use of a chemotherapeutic agent and radiotherapy improves survival in patients with locally advanced or recurrent cervical cancer, severe side effects related to chemotherapy are frequent and may result in a low quality of life for the patients. In this study, we investigated the effects of a combination of Wee1 inhibitor (AZD1775) and irradiation in cervical cancer*. In vitro* effects of AZD1775 with irradiation in human cervical cancer cells were assessed by clonogenic survival and apoptosis assays. The effects on DNA damage response signaling and the cell cycle were also explored. Tumor growth delay was evaluated to investigate the *in vivo* effects of AZD1775 with irradiation in cervical cancer mouse models, including xenografts and patient-derived xenografts (PDXs). The co-treatment of AZD1775 and irradiation significantly decreased clonogenic survival and increased apoptosis in cervical cancer cells. These effects were associated with G2 checkpoint abrogation which resulted in persistent DNA damage. Both in the xenografts and the PDXs, the co-treatment significantly decreased tumor growth compared tothe irradiation alone (*p* < 0.05). These results demonstrate that the Wee1 inhibitor (AZD1775) can be considered as a potential alternative as a radiosensitizer in cervical cancer instead of a chemotherapeutic agent such as cisplatin.

## Introduction

Concurrent chemoradiation (CCRT) is a standard treatment for locally advanced or recurrent cervical cancer. Cisplatin-based CCRT has been shown to reduce the risk of recurrence and death by 30–50% compared to radiation therapy (RT) alone by increasing local control rates. Results from five large randomized trials comparing cisplatin-based CCRT against RT alone indicate that cisplatin exerts its antitumoral effects as a radiosensitizer^1^. Although CCRT is tolerable in most patients, the addition of cisplatin to irradiation may cause acute toxicities such as nephrotoxicity and ototoxicity. It has also been associated with a higher rate of hematologic and gastrointestinal adverse effects compared to RT alone^2,3^. These adverse effects are the major limitation of CCRT in clinical practice.

Rendering a cell unable to repair double-strand breaks (DSBs) induced by ionizing radiation (IR) leads to cell death^4^. Therefore, it is reasonable to combine the inhibitors of DNA damage repair mechanisms, such as checkpoint inhibitors, with IR to enhance treatment effects. p53 is well known not only as a cell cycle regulator at the G1 checkpoint that arrests cell cycles thus providing time for a cell to repair damage, but also as an activator of genes that are directly involved in DNA damage repair response^5^. As a result, for tumors in which the G1 checkpoint is bypassed by p53 inactivation, the DNA repair system may become solely dependent on the G2 checkpoints. It has indeed been shown that Wee1, the G2 checkpoint, is upregulated in p53-mutated cancer cells. The development of cervical cancer involves functional p53 inactivation by human papillomavirus (HPV) infection. More than 90% of patients with cervical cancer showed HPV E6-mediated inactivation of p53 in their primary tumors^6,7^, suggesting that the G2 checkpoints as a potential treatment target in cervical cancer.

Many clinical trials have tested the ability of the Wee1 inhibitor AZD1775 to impair the growth of different types of cancer alone or in combination with other cytostatic agents (*e.g*., cisplatin, paclitaxel, 5-fluorouracil, and topotecan)^8^. In this context, the co-treatment with a Wee 1 inhibitor, which is a G2 checkpoint inhibitor, with IR for cervical cancer may have a significant dampening effect on the recovery of cancer cells from the RT-induced DNA damage that may eventually lead to cell death. This study was designed to investigate the preclinical efficacy of the combination therapy of the Wee1 inhibitor and IR in cervical cancer models. Our data show for the first time that Wee1 inhibition by AZD1775 abrogates the prolonged the G2 checkpoint induced by RT and leads to dramatic radiosensitization in cervical cancer.

## Results

### Wee1 expression and effect of AZD1775 with IR in cervical cancer cells

**Figure 1:**
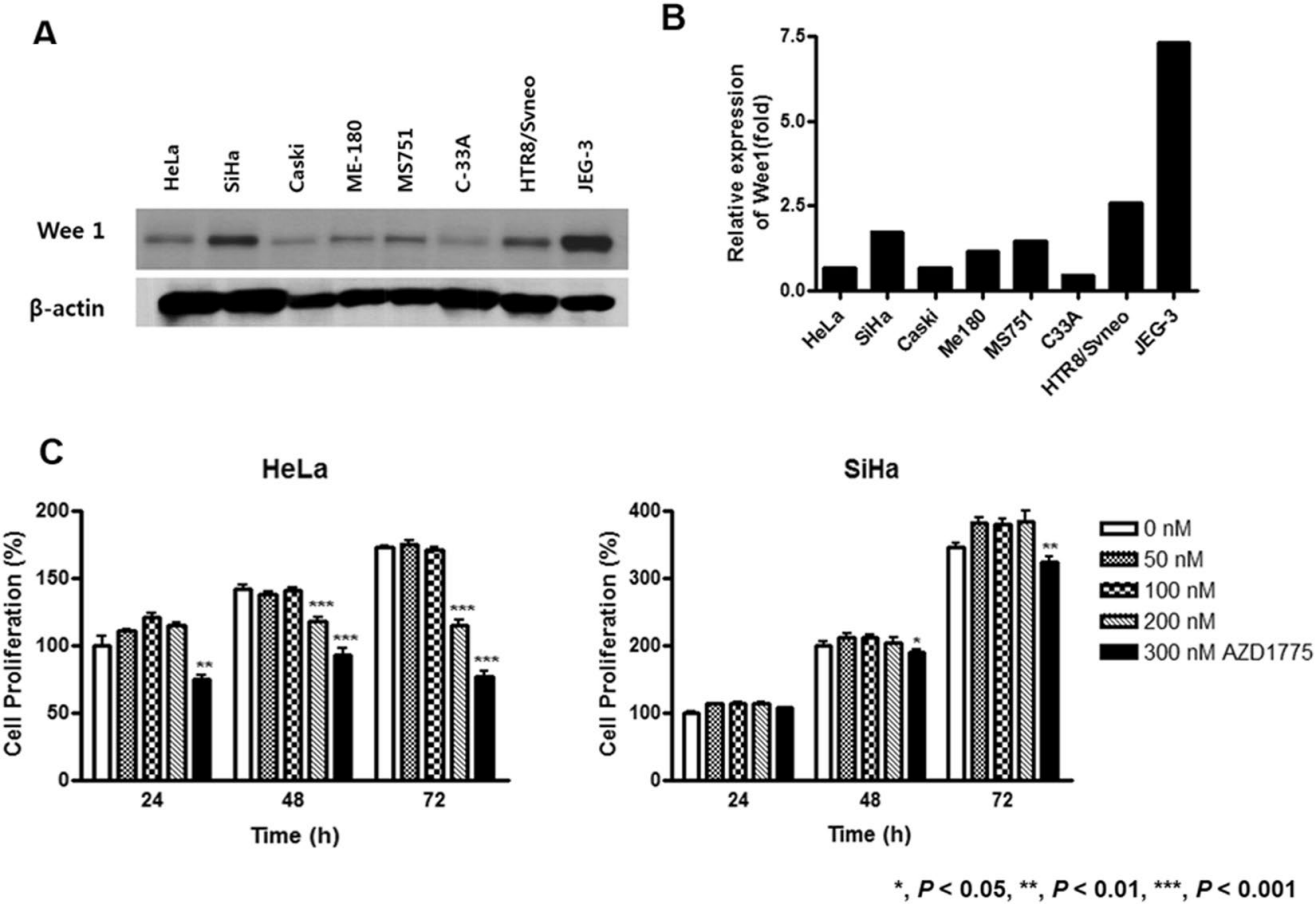
Wee1 expression in cervical cancer cell lines and cytotoxic effects of AZD1775 in HeLa and SiHa cells. Cervical cancer cell lines showed lower intensities of Wee1 expression compared to trophoblastic cell lines (**A**). Quantification of Wee1 expression (**B**). Cell proliferation assay of AZD1775 (**C**). All experiments were repeated three times. The error bar represents the standard error of the mean (s.e.m.).

Cervical cancer cell lines showed lower intensities of Wee1 expression as opposed to placental trophoblastic cells (Fig. 1A,B). To examine the effect of AZD1775 on cell proliferation, we performed the MTT assay in HeLa and SiHa cells treated with various concentrations of AZD1775 (Fig. 1C). In HeLa cells, AZD1775 did not affect cell survival at 50 nM and 100 nM at 24, 48, and 72 h, whereas survival decreased at 200 nM and 300 nM. In SiHa cells, cell survival was not influenced by AZD1775 at 50, 100, and 200 nM but was inhibited by 300 nM at 48 and 72 h. In C-33A cells, AZD1775 showed a similar effect on cell proliferation demonstrated in HeLa and SiHa cells (Supplementary Fig. 1).

**Figure 2:**
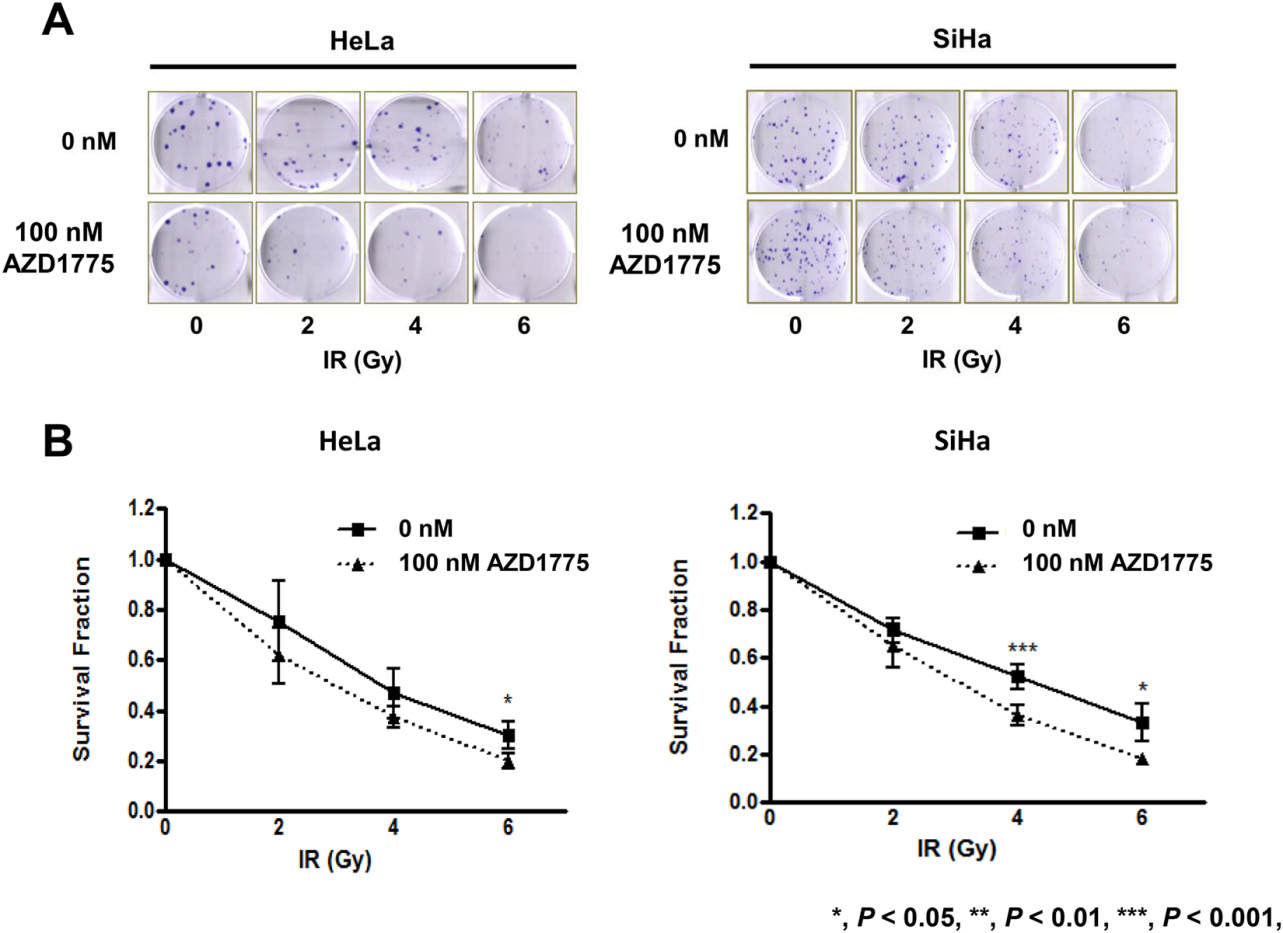
Clonogenic assay for AZD1775 and IR treatment in HeLa and SiHa cells. HeLa and SiHa cells were treated with 100 nM AZD1775 1 h prior to IR (0, 2, 4, or 6 Gy) and the number of clones were counted 12 days after IR (**A**). A clonogenic survival analysis was also performed (**B**). All experiments were repeated three times. The error bar represents standard error of the mean (s.e.m.).

In clonogenic survival analysis, AZD1775 enhanced the effects of IR on the survival fraction in both HeLa and SiHa cells (Fig. 2). AZD1775 was added 1 h prior to IR to determine whether Wee1 inhibition enhanced radiosensitization. With a non-toxic concentration of AZD1775 (100 nM), we observed a modest to significant clonogenic survival effect with IR in both cell lines. AZD1775 at 100 nM showed a significantly decreased colony number with 6 Gy IR in HeLa cells, and with 4 Gy and 6 Gy in SiHa cells (Fig. 2B).

### The effect of AZD1775 pretreatment on apoptosis in cells treated with IR

**Figure 3:**
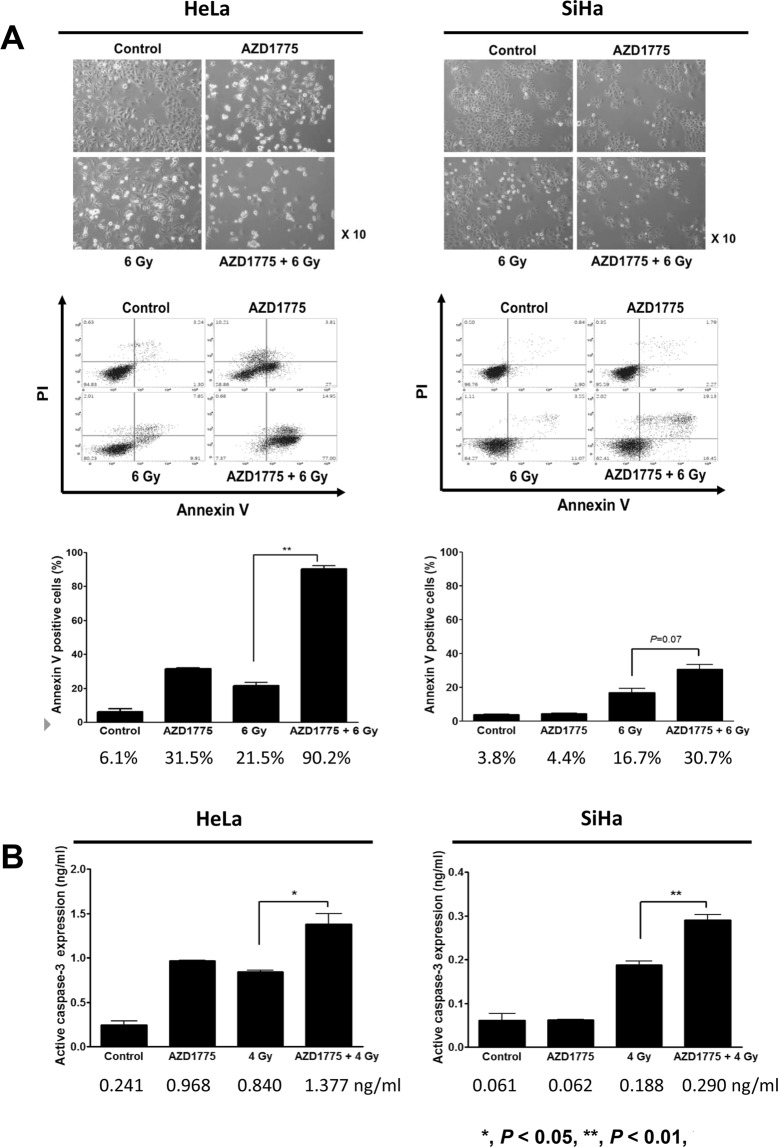
AZD1775 and IR enhanced apoptosis in HeLa and SiHa cells. Cells were treated with 100 nM AZD1775 1 h prior to IR (6 Gy). At 72 h after IR, light microscopy photographs were taken (×10) and annexin V was quantified by FACS analysis for each group (**A**). Apoptosis assay was performed by measuring active caspase-3 with ELISA. AZD1775 (100 nM) was added 1 h prior to IR (4 Gy) and active caspase-3 was measured 48 h after IR (**B**). All experiments were repeated three times. The error bar represents standard error of the mean (s.e.m.).

We investigated the effects of combined treatment of Wee1 inhibitor with IR on apoptosis in cervical cancer cells by annexin V immunostaining and FACS analysis. When cells were pretreated with 100 nM AZD1775 1 h before IR at 6 Gy, the apoptosis activity at 72 h after IR was significantly higher in HeLa cells (AZD1775 + 6 Gy *vs*. 6 Gy, 90.2% *vs*. 21.5%, *p* < 0.001) and there was a modest increase in SiHa cells (AZD1775 + 6 Gy *vs*. 6 Gy, 30.7% *vs*. 16.7%, *p* = 0.07), as shown in Fig. 3A.

To confirm the effects of co-treatment on apoptosis, we measured the expression of active caspase-3 in both cell lines by ELISA. Cells were treated with 100 nM AZD1775 1 hour prior to IR with 4 Gy and active caspase-3 was measured 48 h after IR. Pretreatment with AZD1775 significantly increased the apoptotic effect of IR in both cell lines (Fig. 3B).

### Influence of AZD1775 and IR on DNA damage response and cell cycle in cervical cancer cells

**Figure 4:**
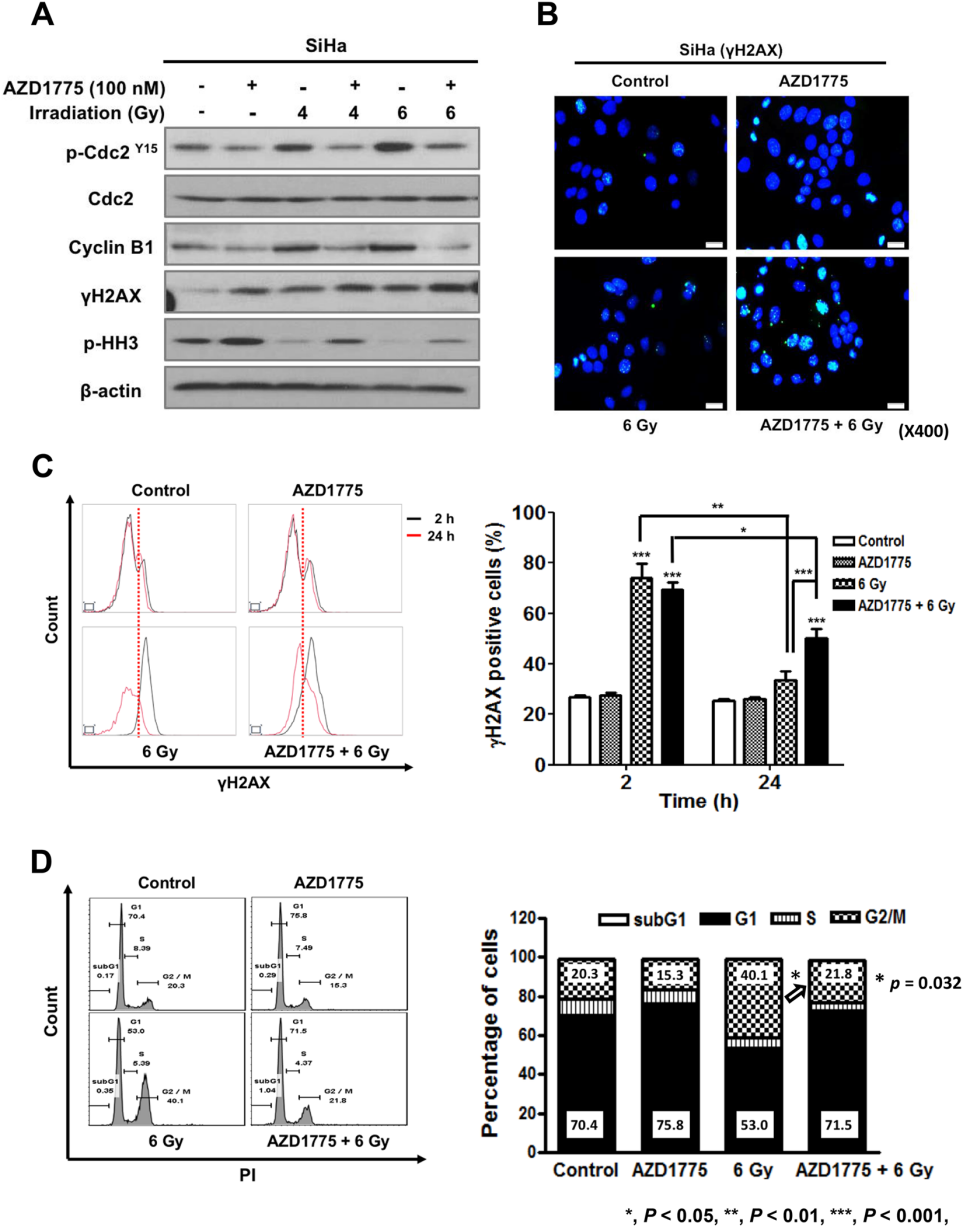
The effects of AZD1775 and IR on DNA damage response signaling and cell cycle distribution. SiHa cells were treated with 100 nM AZD1775 1 h prior to IR (4 or 6 Gy). At 24 h after treatment, cells were analyzed by immunoblotting for the indicated proteins (**A**). Immunocytochemistry was performed with γH2AX in SiHa cells. Cells were pretreated with 100 nM AZD1775 1 h prior to IR (6 Gy). At 24 h after IR, cells were stained for γH2AX (green) and with DAPI (blue). Images are from a single representative experiment (**B**). SiHa cells were treated with 100 nM AZD1775 1 h prior to IR (6 Gy) and then analyzed for γH2AX by flow cytometry at 2 h and 24 h after IR (**c**). Cell cycle distribution in SiHa cells exposed to the same treatment was analyzed by FACS. The percentage of cells in each phase of the cell cycle is indicated (**D**). All experiments in (**C**,**D**) were repeated three times. The error bar represents standard error of the mean (s.e.m.).

To investigate the mechanism of the combination effect of AZD1775 and IR on cell proliferation, we investigated the downstream targets of Wee1 kinase in SiHa cells. After 24 h from IR with 4 or 6 Gy, western blotting was performed to investigate the effects of AZD1775 on phosphorylated-Cdc2 (pCdc2) and Cyclin B1. The co-treatment of AZD1775 (100 nM) with IR reduced the expression of pCdc2 and Cyclin B1 compared to IR alone (Fig. 4A and Supplementary Fig. 2). Similar patterns of expressions were observed in SiHa and C-33A (Supplementary Figs 3–6). Expression of γH2AX, an established marker of DNA DSBs^9^, was increased after the co-treatment compared to IR alone. The level of phosphorylated histone H3 (pHH3), a marker of mitotic activity^10^, decreased in cells at 24 h after IR in the absence of AZD1775. The addition of AZD1775 prior to IR increased pHH3 expression, suggesting that cells with damaged DNA from IR were forced to enter mitosis by the inhibition of Wee1 (Fig. 4A). We confirmed the increased expression of γH2AX in the co-treatment group of SiHa cells using immunocytochemistry and flow cytometry (Fig. 4B,C, and Supplementary Fig. 7). The same results were found in HeLa and C-33A cells (Supplementary Figs 8 and 9).

To further investigate the mechanisms of combined AZD1775 with IR, we assessed the effects on the cell cycle (Fig. 4D). We hypothesized that AZD1775-mediated Wee1 inhibition would abrogate the IR-induced G2 checkpoint. First, we investigated the quantification of IR-induced DNA damage at two time points (2 h and 24 h after IR). At 2 h after IR, DNA damage was significantly increased regardless of presence of AZD1775. However, at 24 h, the level of IR-induced DNA damage with co-treatment resembled that of cells without IR or the pretreatment of AZD1775 (Fig. 4C). Of note, the cells pretreated with AZD1775 showed persistent elevation of γH2AX expression at 24 h after IR, suggesting the impairment of the G2 checkpoint, which provides cells time to repair DNA damage. In line with these results, we observed the abrogation of the IR-induced G2 checkpoint in the presence of AZD1775, as evidenced by the increase in the percentage of cells in G1 and corresponding decrease in the percentage of cells in G2 (Fig. 4D). The same results were found in HeLa and C-33A cells (Supplementary Fig. 10).

### *In vivo* effect of AZD1775 with IR in SiHa cell xenografts

**Figure 5:**
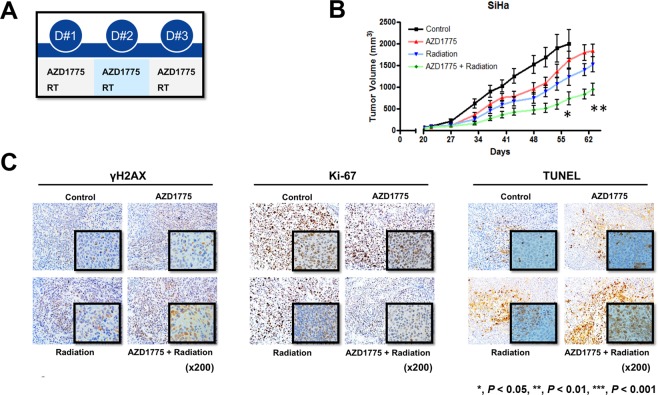
Combination effect of AZD1775 and IR in SiHa cell xenografts. Athymic nude mice bearing SiHa xenografts in their flanks were treated with AZD1775 (60 mg/kg; oral, one a day; 1 h before IR for 3 days) and IR (2 Gy/fraction for 3 days), as illustrated (**A**). Treatment started when the average tumor volume reached 100 mm^3^ in all groups. Tumor volume (TV) was calculated according to the equation: TV = π/6 (ab^2^), where a and b are the long and short dimensions of the tumor. Mice were euthanized at 120 days post tumor injection, or when tumor volume reached at 1000 mm^3^, or when the mice were in a moribund condition, whichever occurred first. Tumors were excised at the time of euthanasia (**B**). Immunostaining of γH2AX and Ki-67 and TUNEL assay (×200) were performed (**C**). The error bar represents standard error of the mean (s.e.m.).

Mice bearing subcutaneous SiHa tumor xenografts were treated with AZD1775 daily (60 mg/kg; per oral, once a day; 1 h before IR) and IR (three fractions with 2 Gy each), as depicted in Fig. 5A. In the absence of IR, AZD1775 had a modest delaying effect on tumor growth (Fig. 5B). AZD1775 in combination with fractionated IR induced a significant delay in tumor growth relative to IR alone (*p* < 0.05). At the time of euthanasia, we performed immunohistochemical evaluation of tumor samples in each group. The expression of γH2AX in tumor samples was increased in mice in the co-treatment group compared to the other three groups. The co-treatment also decreased cell proliferation assessed by Ki-67 expression and increased apoptosis assessed by TUNEL assay in the co-treatment samples compared to the others (Fig. 5C). Systemic toxicities leading to moribund state, fit criteria for euthanasia, or death were not observed during any type of treatments across the four groups.

### Effect of AZD1775 with IR in PDX models of cervical cancer

**Figure 6:**
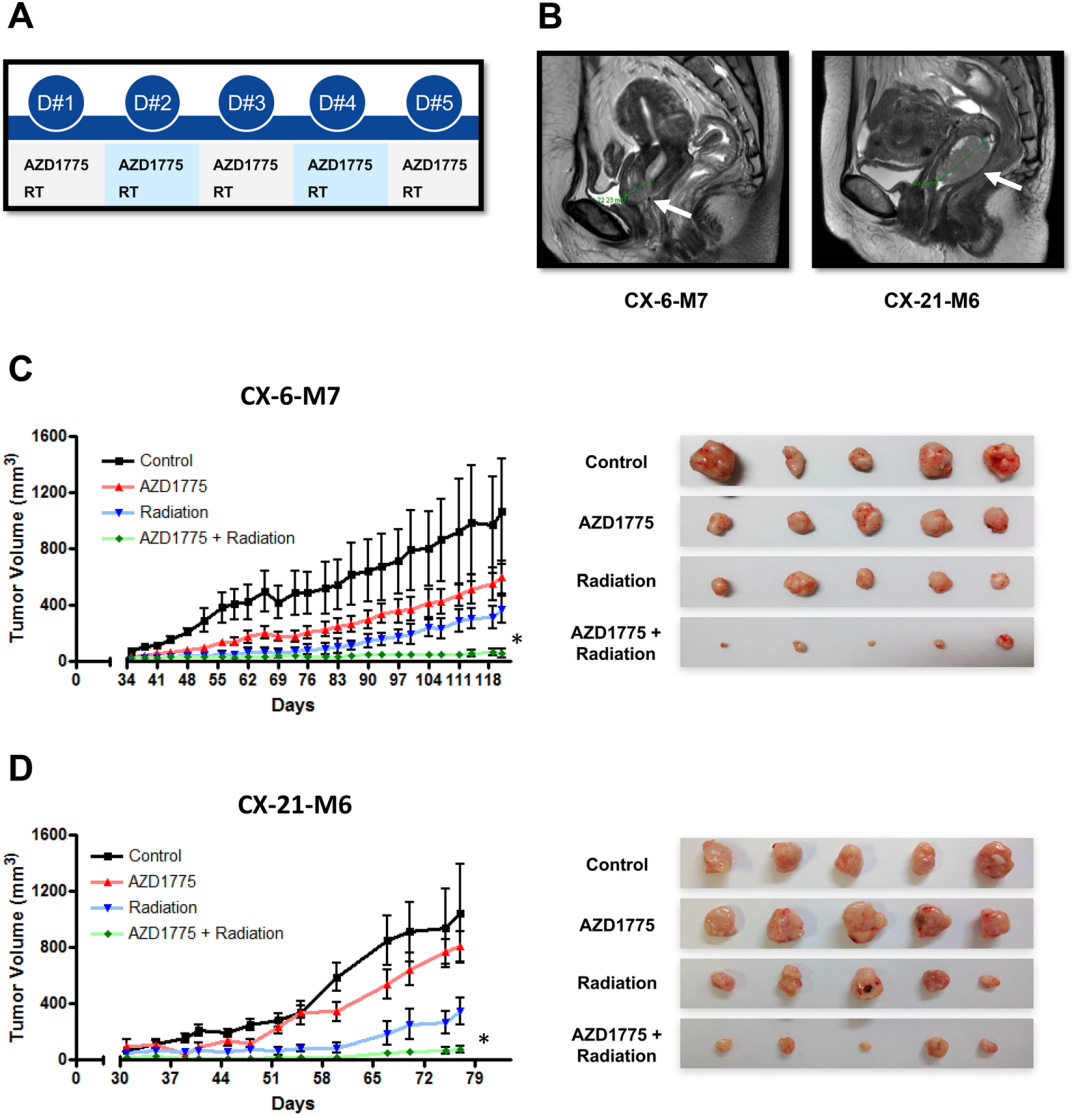
Combination effect of AZD1775 and IR in cervical cancer PDX models. Athymic nude mice bearing PDX in their flank were treated with AZD1775 (60 mg/kg; oral, once a day; 1 h before IR for 5 days) and radiation (RT; 1.8 Gy/fraction for 5 days) as illustrated (**A**). T2-weighted sagittal MRI shows a 2.2-cm-sized cervical tumor (left white arrow) in the anterior lip of the cervix and a 4.9-cm-sized cervical tumor (right white arrow) occupying the upper vaginal canal (**B**). Treatment started when the average tumor volume reached 100 mm^3^ in all groups. Tumor volume (TV) was calculated according to the equation: TV = π/6 (ab^2^), where a and b are the long and short dimensions of the tumor. Mice were euthanized at 120 days post tumor injection, or when the tumor volume reached 1,000 mm^3^, or when the mouse was in a moribund condition, whichever occurred first. Tumors were excised at the time of euthanasia (**C**). The error bar represents standard error of the mean (s.e.m.).

*In vivo* experiments using PDXs were carried out with a more fractionated protocol. Our group previously established and reported a series of PDX models for cervical cancer^11^. We selected two currently available PDX models for the present study. In these models, IR was given in five fractions at a dose of 1.8 Gy/fraction because the treatment plan used in SiHa cell xenografts (three fractions with 2 Gy/fraction) did not effectively decrease tumor growth in PDX models. CX-6-M7 and CX-21-M6 were tumors derived from patients with early (FIGO stage IB1) and locally advanced (FIGO stage IB2) cervical cancer, respectively. Histology revealed invasive squamous cell carcinoma with high-risk HPV (+) in both cases. Pretreatment with AZD1775 was carried out 1 h prior to IR. In the absence of IR, AZD1775 had a modest delaying effect on tumor growth (Fig. 6). AZD1775 in combination with fractionated IR significantly inhibited tumor growth rates compared with IR or AZD1775 alone in both models (*p* < 0.05). Systemic toxicities leading to moribund state, fit criteria for euthanasia, or death were not observed during any type of treatments across the four groups.

## Discussion

Due to the potential toxicity from chemotherapeutic agents, replacing chemotherapy in CCRT with targeted agents is an attractive alternative for treatment of cancer types that are usually treated with RT, such as head and neck, cervical, and anal cancers. In this study, we showed that the inhibition of Wee1 by AZD1775 significantly increased the efficacy of RT by acting as a radiosensitizer in cervical cancer models. Mechanistically, AZD1775 abrogates the prolonged G2 checkpoint induced by RT in cervical cancer cells, causing DNA damaged cells to enter M phase and subsequently undergo apoptosis. Moreover, in SiHa cell xenografts and two PDX models for cervical cancer, the combination treatment with AZD1775 and RT induced significant tumor growth delay compared with radiation alone."

Inhibition of Wee1, which is known to be overexpressed in HPV-positive head and neck squamous cell carcinoma (HNSCC), has been shown to sensitize tumor cells to cytotoxic agent such as cisplatin^12–15^. Because Wee1 is essential for cell survival in p53-mutant HNSCC cells, the treatment with AZD1775 leads to the death of those tumor cells. The fact that both HPV-positive and -negative tumor cells responded similarly to AZD 1775 implicates that the mutational status of p53, rather than the status of HPV infection, is responsible for preferential sensitivity to Wee1 inhibitors^16^.

We could not find any changes in the expression of Wee1following the treatment of AZD1775. In designing the experiments, we chose the sublethal doses of AZD1775, which would better show its potential role as a radiosensitizer. This would explain, in part, the reason for the unchanged Wee1 expression observed. For example, in a pancreatic cancer preclinical model, the same doses of AZD1775 that were used in the present experiments also did not change the Wee1 expression while showing the effect of radiosensitizer by reducing pCdk1^17^. In addition, cells of uterine cervix are reported to show lower Wee1 expression levels compared to other parts of the body including placenta^18^. Due to its relatively low Wee1 expression, it might be difficult to see any differences of Wee1 expression with relatively low doses of Wee1 inhibitor. However, this would not necessarily be of concern when examining the effects of Wee1 inhibitor because the inhibition of Wee1 has been shown to activate Cdk1, thus abrogating the radiation-induced G2 checkpoint. This would lead to impaired homologous recombination repair^19^, and induce replication stress^20^.

The mutational status of p53 seems to be related to the preferential response of tumor cells to Wee1 inhibitors^21–23^. Studies have shown that AZD1775 radiosensitized p53-defective human cells derived from lung, breast, and prostate cancers by abrogating the radiation-induced G2 block, while this was not observed in p53 wild-type cells^24^. However, studies showing contrast results exists as well. Sensitization of Hep3B, Huh7, and HepG2 cell lines to radiotherapy and chemotherapy was demonstrated following the treatment with AZD1775 in hepatocellular carcinoma regardless of their p53 mutational status^25^. The combination of AZD1775 and Chk1 inhibitors (LY2603618 and MK8776) was shown to radiosensitize HPV-positive HNSCC cells, suggesting a potential use in de-intensified regimes^26^.

The single agent AZD1775 showed tolerable toxicity in a phase I study in patients with refractory solid tumors, in which common toxicities were reported to be myelosuppression and diarrhea^27^. In this study, we found no significant difference in terms of systemic toxicities between treatment and control groups in all xenograft experiments. This suggests that the addition of Wee1 inhibitor did not produce serious complications in mice treated with IR compared with controls; however, it will be necessary to evaluate the complications of Wee1 inhibition in RT-induced normal tissue in further studies.

We also did not find any differences in the levels of Wee1 protein expression with regard to the treatment of AZD1775 (Fig. 1A) in cell proliferation tests. We suggest that the effect of Wee1 inhibition is rather dependent on the functional status of p53, and not on the levels of Wee1 expression.

Further studies are still warranted in order to incorporate targeted agents with IR in replacement of the conventional regimens.

In this study, we showed for the first time that Wee1 inhibition by AZD1775 abrogated the prolonged G2 checkpoint induced by RT and led to dramatic radiosensitization in cervical cancer, which was considered to have functional p53 inactivation through HPV infection. Based on our results, AZD1775 could be considered as a radiosensitizer in cervical cancer instead of chemotherapeutic drugs like cisplatin. This strategy may also have broader indications for other HPV-related cancers or cancers with p53 mutation in which primary radiation is a standard treatment.

## Methods

### Cell culture

Human cervical cancer cell lines (HeLa, SiHa, ME-180, MS751, Ca Ski, and C-33A) and placental trophoblastic cell lines (HTR8/Svneo, JEG-3) were obtained from the American Type Culture Collection (American Type Culture Collection, Manassas, VA, USA). The HeLa cell line was cultured in DMEM (Gibco BRL, Gaithersburg, MD, USA); SiHa, ME-180, MS751, and C-33A cell lines were cultured in MEM (Gibco BRL); and the Ca Ski cell line was cultured in RPMI 1640 (Gibco BRL). All media were supplemented with 10% fetal bovine serum (FBS; Sigma-Aldrich, St. Louis, MO, USA) and 0.1% penicillin-streptomycin (Sigma-Aldrich). All cells were grown at 37 °C in a humidified 5% CO_2_ atmosphere.

### Reagents and irradiation (IR)

AZD1775 was obtained from AstraZeneca (AstraZeneca, Waltham, MA, USA). For *in vitro* experiment, dimethylsulphoxide (DMSO) at 10 µM was used for vehicle and control. IR was performed using a Varian Clinac 6EX linear accelerator (Varian Medical Systems, Palo Alto, CA, USA). Cell monolayers were treated with various doses of 6 MV photons at a rate of 3.96 Gy/min. The distance between the IR source and the cell plates were kept at 100 cm while the field size was fixed at 30 × 30 cm. Cell plates were placed under a 2-cm-thick solid water phantom. The absolute photon dose was calibrated according to TG-51 and verified with Gafchromic film to 1% accuracy.

### Western blot analysis

The expression of Wee1 in human cervical cancer cell lines was examined by western blot analysis. SiHa cells were seeded in a 6-well plate (2 × 10^5^ cells/well) a day before IR. Cells were pretreated with AZD1775 (100 nM) for 1 h and then exposed to IR at doses of 4 Gy and 6 Gy^17^. After 24 h, cells were lysed in PRO-PRE Protein Extraction Solution (Intron Biotechnology, Seongnam, South Korea) according to the manufacturer’s protocol. Total proteins were separated by SDS-PAGE and transferred to a hydrophobic immobilon-P PVDF Transfer Membrane (Millipore, Billerica, MA, USA). Membranes were blocked with 5% BSA in Tris-buffered saline containing 0.1% Tween-20 for 1 h at room temperature. Antibodies used for protein bands probe were anti-Wee1 antibody (Santa Cruz Biotechnology, Santa Cruz, CA, USA), anti-phospho-γH2AX (Ser139) antibody (Cell Signaling Technology, Beverly, MA, USA), anti-phospho-cdc2 (Tyr15) antibody (Cell Signaling Technology), anti-cdc2 antibody (Cell Signaling Technology), anti-cyclin B1 antibody (Epitomics, Burlingame, CA, USA), anti-phospho-Histone H3 (Ser10) antibody (Cell Signaling Technology), and anti-β-actin antibody (Santa Cruz Biotechnology). Then they were labeled with horseradish peroxidase-conjugated anti-mouse and anti-rabbit secondary antibody (Sigma-Aldrich). Bands were visualized by enhanced chemoluminescence using an ECL kit (Amersham Biosciences, Buckinghamshire, UK) according to the manufacturer’s protocol.

### 3-(4, 5-dimethylthiazol-2-yl)-2, 5-diphenyl tetrazolium bromide (MTT) assay

For cell proliferation assay, HeLa and SiHa cells were seeded in a 96-well microplate (3 × 10^3^ cells/well), treated with varying concentrations of AZD1775, and incubated at 37 °C for 24, 48, and 72 h. After 4 h of incubation with 3-(4, 5-dimethylthiazol-2-yl)-2, 5-diphenyl-tetrazoliumbromide (MTT) solution (Amresco, Solon, OH, USA), the cells were allowed to mix with 100 µl of acidic isopropanol (0.1 N HCl in absolute isopropanol). Absorbance was measured on an enzyme-linked immunosorbent assay (ELISA) reader at a test wavelength of 540 nm.

### Clonogenic survival assay

Radiosensitivity was determined by clonogenic survival assays as mentioned in previous literature^28^. HeLa and SiHa cells were seeded in a 6-well plate (70–840 cells/well) a day before IR. Cells were pretreated with AZD1775 (100 nM) for 1 h and then exposed to doses of 2, 4 and 6 Gy of IR^17^. After 12 days, cells were stained with 0.1% crystal violet (with 20% methanol) and the number of colonies grown were counted. Colonies with >50 cells were considered viable. Plating efficiency was calculated by dividing the numbers of viable colonies by the number of plated cells. Survival fraction was calculated by dividing the plating efficiency of the irradiated cells by the plating efficiency of the untreated control (DMSO). The samples were measured in triplicate.

### Enzyme-linked immunosorbent assay (ELISA)

HeLa and SiHa cells were seeded in a 6-well plate (2 × 10^5^ cells/well) a day before IR. Cells were pretreated with AZD1775 (100 nM) for 1 h and then exposed to IR at a dose of 4 Gy^17^. After 24 h or 48 h, cells were lysed in PRO-PRE Protein Extraction Solution (Intron Biotechnology) according to the manufacturer’s protocol. ELISA kits were used as described by the manufacturer (Invitrogen, San Diego, CA, USA) to measure the concentrations of human active caspase-3. The samples were measured in triplicate.

### Apoptosis assay

The extent of apoptosis was evaluated by annexin V-FITC staining and flow cytometry. Cells were plated in 6-well plates (2 × 10^5^ cells/well) and allowed to attach overnight. Cells were pretreated with AZD1775 (100 nM) for 1 h and then subjected to 6 Gy IR^17^. After incubation for 72 h, cells were treated with trypsin, washed with PBS (pH 7.4), and stained with anti-annexin V-FITC antibody (BD Pharmingen, San Diego, CA, USA) and 2 μg/ml propidium iodide (PI) in 100 μl annexin V binding buffer (10 mM HEPES, pH 7.4, 140 mM NaCl, 2.5 mM CaCl_2_) for 15 min at 37 °C in the dark. Samples were analyzed by flow cytometry using a BD FACSVerse flow cytometer (Becton-Dickinson, San Jose, CA, USA). The samples were measured in triplicate. Data were acquired using BD FACSuite software.

### Cell cycle analysis

Cell cycle analysis was performed by flow cytometry using PI staining. Cells were plated in 6-well plates (3 × 10^5^ cells/well) and allowed to attach overnight. Cells were pretreated with AZD1775 (100 nM) for 1 h and then exposed to IR for 24 h^17^. Then they were collected by trypsinization, fixed in cold 70% ethanol, washed in PBS, and resuspended in 1 ml of PBS containing 1 mg/ml RNase and 50 g/ml PI. After incubation in the dark for 30 min at 37 °C, cells were analyzed using a FACSVerse flow cytometer. The samples were measured in triplicate.

### DNA damage and repair analysis

IR-induced DNA damage was quantified by two independent methods: immunofluorescence staining and flow cytometry. For immunofluorescence, cells seeded on cover glasses were pre-treated with AZD1775 (100 nM) for 1 h and irradiated with 6 Gy IR^17^. After incubation for 24 h, cells were fixed with 4% formaldehyde and permeabilized with 0.5% Triton X-100. After blocking with 3% BSA for 1 h, cells were incubated with anti-phospho-γH2AX (Ser139) for 2 h, followed by Alexa Fluor 488-conjugated secondary antibodies (Invitrogen, Carlsbad, CA, USA) for 1 h and DAPI (Sigma-Aldrich, St. Louis, MO, USA) for 5 min. Cells were washed, mounted on glass slides using glycerol, and imaged using a fluorescence microscope (Olympus BX51 Microscope, Olympus corporation, Shinjuku, Tokyo, Japan). For flow cytometry, 2 × 10^5^ cells were plated in 6-well plates and allowed to attach overnight. Cells were pretreated with 100 nM AZD1775 for 1 h and irradiated with 6 Gy IR^17^. At 2 h or 24 h after irradiation, cells were collected by trypsinization and fixed in 4% formaldehyde for 10 min followed by permeabilization with 0.01% Triton X-100 for 3 min. Cells were blocked in 2% FBS in PBS for 30 min at room temperature and then incubated with anti-phospho-γH2AX for 1 h. Secondary antibodies were added for 30 min. Data were acquired with BD FACSVerse and analyzed with BD FACSuite Software. Negative controls were stained with secondary antibodies alone.

### Immunohistochemical analysis

Formalin-fixed, paraffin-embedded, 4 µm thick tissue sections were used for immunohistochemical analysis. The primary antibodies used were anti-phospho-γH2AX (Ser139) (Novus Biologicals, Littleton, CO, USA) and anti-Ki-67 (Novus Biologicals). Tissue sections were deparaffinized three times in xylene for a total of 15 min and rehydrated. Immunostaining was done using a Bond-max^TM^ Polymer Refine Detection kit (Vision Biosystems, Melbourne, Australia). Antigen retrieval was performed at 97 °C for 20 min in ER1 buffer. Endogenous peroxidase activity was blocked with 3% hydrogen peroxidase for 10 min and the sections were incubated with primary antibody for 15 min at room temperature at an antibody dilution of 1:200. Anti-rabbit IgG (Vector Laboratories, Burlingame, CA) was used in place of the primary antibody as a negative control.

### TUNEL assay

A terminal deoxynucleotidyl transferase-mediated dUTP nick and labeling (TUNEL) assay was performed for the assessment of apoptotic cell death. Deparaffinization, rehydration and blocking of endogenous peroxidase with 3% hydrogen peroxide in PBS for 10 min at room temperature was done. The digestion of the tissue sections with 20 μg/mL proteinase K in PBS was then performed for 15 min at room temperature. PBS buffer washing was treated, followed by the treatment of equilibration buffer at room temperature. Then the incubation with working strength terminal deoxynucleotidyl transferase (TdT) for 60 min at 37 °C in a humidity chamber was carried out. The termination of the reaction was performed with working strength stop/wash buffer for 30 min at room temperature. Covering the sections ith anti-digoxigenin-peroxidase was done for 30 min at room temperature after washing in PBS. Then DAB substrate chromogen solution was applied for the development of color reaction for 5 min. Finally, the sections were counterstained with Mayer’s hematoxylin for 30 sec for after washing with distilled water. H & E stains were done for all sections for histologic evaluation as well.

### Animal care and development cell line and patient-derived xenografts (PDXs) as *in vivo* models

Female BALB/c nude mice were used (Orient Bio, Seongnam, Korea). To develop cell line xenografts, SiHa cells (1 × 10^6^ cells/0.1 mL HBSS) were injected subcutaneously into the right hind legs of mice. For PDX development, patient tumor was surgically removed and were sliced to small pieces (less than 2–3 mm). Then they were implanted subcutaneously into the right hind leg of mice. The PDXs were maintained by serial transplantation^11^. PDXs were considered to be successfully established after three passages with a stable growth pattern^11^.

When the mean tumor volume reached 80–100 mm^3^, mice with cell line xenografts or PDXs were randomized to one of four groups (10 mice per treatment group): control (0.5% methylcellulose in PBS), AZD1775, irradiation, or AZD1775 + irradiation. AZD1775 (60 mg/kg once a day in 0.5% methylcellulose solution, 1 h pre-IR) was administered orally over a 3–5 day period. The doses and schedules of administration of AZD1775 and irradiation were modified from a previously published study for the present study^17^. We used the half doses of AZD1775 in total compared to those used in the previous study in which no systemic toxicity was reported. An X-ray beam was directed on the tumor-bearing right hind leg of the mice (for cell line xenograft model: 2 Gy/d × 3 days; for PDX model: 1.8 Gy/d × 5 days). During IR, mice were anesthetized by intraperitoneal injection of 30 mg/kg zolazepam/tiletamine and 10 mg/kg xylazine under the prescription of a veterinarian. Tumor volumes were measured twice a week with calipers. Tumor volume (TV) was calculated according to following equation: TV = π/6 (ab^2^), where a and b are the longer and shorter dimensions of the tumor^17^. Mice were sacrificed when the tumor volume reached a dimension of at least 2,000 mm^3^ or when the mouse developed clinical signs^29^. Tumors were fixed in formalin and embedded in paraffin or snap frozen in OCT compound (Sakura Finetek Japan, Tokyo, Japan) in liquid nitrogen.

This study was conducted with approval of the Samsung Medical Center Institutional Review Board (IRB File No. 2010-04-004-121) and carried out in accordance with approved guidelines and regulations. We used “passage 7” (M7) of CX-6 model and “passage 6” (M6) of CX-21 in our PDX library for this experiment. The study was also reviewed and approved by the Institutional Animal Care and Use Committee (IACUC) of Samsung Biomedical Research Institute (protocol No. 20161110002), which is an Association for Assessment and Accreditation of Laboratory Animal Care International (AAALAC International) accredited facility and abides by the Institute of Laboratory Animal Resources (ILAR) guidelines.

### Statistical analysis

*The Wilcoxon rank sum-test* and *two-sample t-test* were used to compare median and mean values respectively, after normality test by *the Shapiro-Wilks test*. *The Mann–Whitney U test* or *one-way ANOVA* with the least significant difference post-comparison test was used to evaluate the differences among the groups for *in vitro* and *in vivo* assays. SPSS software (version 17.0; SPSS, Chicago, IL) was used for all statistical analyses. All *p*-values were two-sided and considered statistically significant for *p* < 0.05.

## Supplementary information

Supplementary figures

Supplementary figures legends

Full blots
