# Supplementary material for: Anti-Tumor Effects of Wee1 Kinase Inhibitor with Radiotherapy in Human Cervical Cancer
Source: Sci Rep. 2019 Oct 28;9:15394. doi: 10.1038/s41598-019-51959-3 (PMC6817901; doi:10.1038/s41598-019-51959-3)
Supplement: Supplementary file 2 — Supplementary figures legends [file 41598_2019_51959_MOESM2_ESM.pdf]

Supplementary information

Anti-Tumor Effects of Wee1 Kinase Inhibitor with Radiotherapy in Human Cervical Cancer

Yoo-Young Lee[1,+], Young-Jae Cho[3,+], Sung-won Shin[2,+], Changhoon Choi[2], Ji-Yoon Ryu[3], Hye-Kyung Jeon [3], Jung-Joo Choi[3], Jae Ryoung Hwang[3], Chel Hun Choi[1], Tae-Joong Kim[1], Byoung- Gie Kim[1], Duk-Soo Bae[1], Won Park[2,*], Jeong-Won Lee[1,4,5,*]

[1]Department of Obstetrics and Gynecology, Samsung Medical Center, Sungkyunkwan University School of Medicine, Seoul, Korea

[2]Department of Radiation Oncology, Samsung Medical Center, Sungkyunkwan University School of Medicine, Seoul, Korea

[3]Samsung Biomedical Research Institute, Samsung Medical Center, Sungkyunkwan University School of Medicine, Seoul, Korea

[4]Institute for Refractory Cancer Research, Samsung Medical Center, Seoul, Korea.

[5]Samsung Advanced Institute for Health Sciences & Technology, Sungkyunkwan University School of Medicine, Seoul, Korea

[+]These authors contributed equally to this paper.

Corresponding Author: Won Park, M.D., Ph.D.

Professor

Department of Radiation Oncology, Samsung Medical Center, Sungkyunkwan University School of Medicine, 81 Irwon-ro, Gangnam-gu, Seoul, Korea, 06351

Tel: 82-2-3410-2616; Fax: 82-2-3410-2619; E-mail: wonro.park@samsung.com

Corresponding Author: Jeong-Won Lee, M.D., Ph.D.

Associate Professor

Department of Obstetrics and Gynecology, Samsung Medical Center, Sungkyunkwan University School of Medicine, 81 Irwon-ro, Gangnam-gu, Seoul, Korea, 06351

Tel: 82-2-3410-1382; Fax: 82-2-3410-0630; E-mail: garden.lee@samsung.com

Supplementary Figure 1. Cell proliferation assay for AZD1775 in C-33A cells.

All experiments were repeated three times. The error bar represents standard error of the mean (s.e.m.).

Supplementary Figure 2. The effects of AZD1775 and IR on DNA damage response signaling in SiHa cells. This is quantification of protein expressions of western blot in Figure 4A

Supplementary Figure 3. A, The effects of AZD1775 and IR on DNA damage response signaling in HeLa, SiHa, and C33A. Cells were treated with 100 nM AZD1775 1 h prior to IR (4 or 6 Gy). At 24 h after treatment, cells were analyzed by immunoblotting for the indicated proteins. B, The effect of AZD1775 with various doses on expression of Wee1 in HeLa, SiHa, and JEG-3

Supplementary Figure 4. The effects of AZD1775 and IR on DNA damage response signaling in HeLa cells. Quantification of protein expressions of western blot in supplementary Figure 3

Supplementary Figure 5. The effects of AZD1775 and IR on DNA damage response signaling in SiHa cells. Quantification of protein expressions of western blot in supplementary Figure 2

Supplementary Figure 6. The effects of AZD1775 and IR on DNA damage response signaling in C33A cells. Quantification of protein expressions of western blot in supplementary Figure 2

Supplementary Figure 7. The effects of AZD1775 and IR on DNA damage response signaling. Immunocytochemistry was performed with γH2AX in SiHa cells. Cells were pretreated with 100 nM AZD1775 1 h prior to IR (6 Gy). At 2 h and 24 h after IR, cells were stained for γH2AX (green) and with DAPI (blue). Images are from a single representative experiment (a). HeLa cells were treated with 100 nM AZD1775 1 h prior to IR (6 Gy) and then analyzed for γH2AX by flow cytometry at 2 h and 24 h after IR (b). The error bar represents standard error of the mean (s.e.m.).

Supplementary Figure 8. The effects of AZD1775 and IR on DNA damage response signaling. Immunocytochemistry was performed with γH2AX in HeLa cells. Cells were pretreated with 100 nM AZD1775 1 h prior to IR (6 Gy). At 2 h and 24 h after IR, cells were stained for γH2AX (green) and with DAPI (blue). Images are from a single representative experiment (A). SiHa cells were treated with 100 nM AZD1775 1 h prior to IR (6 Gy) and then analyzed for γH2AX by flow cytometry at 2 h and 24 h after IR (B). The error bar represents standard error of the mean (s.e.m.).

Supplementary Figure 9. The effects of AZD1775 and IR on DNA damage response signaling. Immunocytochemistry was performed with γH2AX in C33A cells. Cells were pretreated with 100 nM AZD1775 1 h prior to IR (6 Gy). At 2 h and 24 h after IR, cells were stained for γH2AX (green) and with DAPI (blue). Images are from a single representative experiment (a). C33A cells were treated with 100 nM AZD1775 1 h prior to IR (6 Gy) and then analyzed for γH2AX by flow cytometry at 2 h and 24 h after IR (b). The error bar represents standard error of the mean (s.e.m.).

Supplementary Figure 10. The effects of AZD1775 and IR on cell cycle distribution. SiHa, HeLa, and C33A cells were treated with 100 nM AZD1775 1 h prior to IR (6 Gy). At 24 h after treatment, cell cycle distributions were analyzed by FACS. The percentage of cells in each phase of the cell cycle is indicated. The error bar represents standard error of the mean (s.e.m.).
