# Supplementary material for: Anti-Tumor Effects of Wee1 Kinase Inhibitor with Radiotherapy in Human Cervical Cancer
Source: Sci Rep. 2019 Oct 28;9:15394. doi: 10.1038/s41598-019-51959-3 (PMC6817901; doi:10.1038/s41598-019-51959-3)

Supplementary information

Anti-Tumor Effects of Wee1 Kinase Inhibitor with Radiotherapy in Human Cervical Cancer

Yoo-Young Lee[1,+], Young-Jae Cho[3,+], Sung-won Shin[2,+], Changhoon Choi[2], Ji-Yoon Ryu[3], Hye-Kyung Jeon [3], Jung-Joo Choi[3], Jae Ryoung Hwang[3], Chel Hun Choi[1], Tae-Joong Kim[1], Byoung- Gie Kim[1], Duk-Soo Bae[1], Won Park[2,*], Jeong-Won Lee[1,4,5,*]

[1]Department of Obstetrics and Gynecology, Samsung Medical Center, Sungkyunkwan University School of Medicine, Seoul, Korea

[2]Department of Radiation Oncology, Samsung Medical Center, Sungkyunkwan University School of Medicine, Seoul, Korea

[3]Samsung Biomedical Research Institute, Samsung Medical Center, Sungkyunkwan University School of Medicine, Seoul, Korea

[4]Institute for Refractory Cancer Research, Samsung Medical Center, Seoul, Korea.

[5]Samsung Advanced Institute for Health Sciences & Technology, Sungkyunkwan University School of Medicine, Seoul, Korea

[+]These authors contributed equally to this paper.

Corresponding Author: Won Park, M.D., Ph.D.

Professor

Department of Radiation Oncology, Samsung Medical Center, Sungkyunkwan University School of Medicine, 81 Irwon-ro, Gangnam-gu, Seoul, Korea, 06351

Tel: 82-2-3410-2616; Fax: 82-2-3410-2619; E-mail: wonro.park@samsung.com

Corresponding Author: Jeong-Won Lee, M.D., Ph.D.

Associate Professor

Department of Obstetrics and Gynecology, Samsung Medical Center, Sungkyunkwan University School of Medicine, 81 Irwon-ro, Gangnam-gu, Seoul, Korea, 06351

Tel: 82-2-3410-1382; Fax: 82-2-3410-0630; E-mail: garden.lee@samsung.com

Figure 1A **Unprocessed images for blots**

Wee1

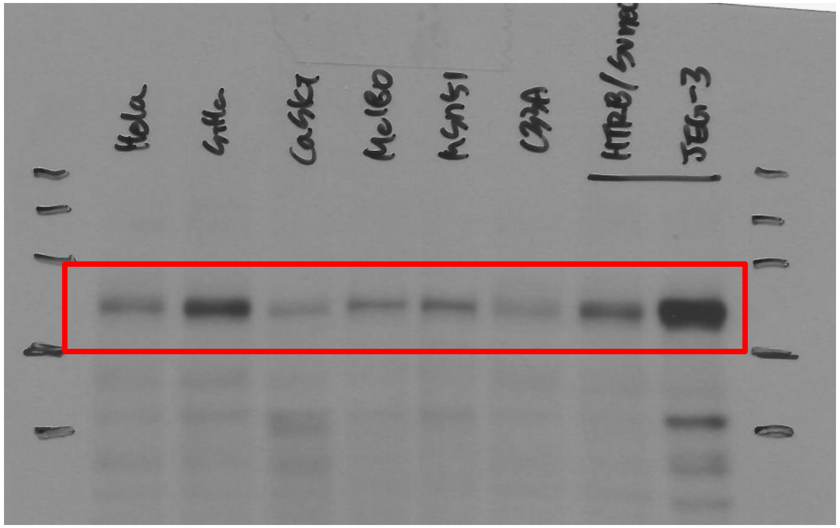

Actin

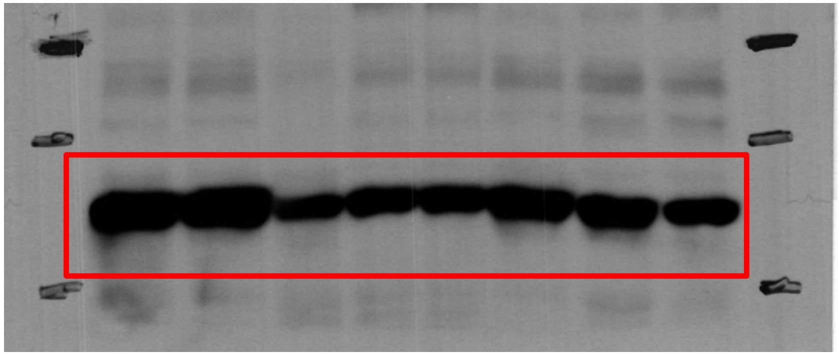

Figure 4A Unprocessed images for blots

γH2AX

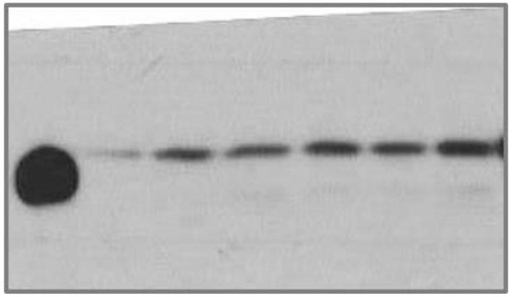

Cdc2

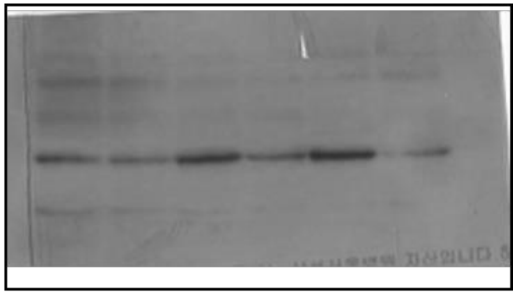

p-cdc2 ^{Y15}

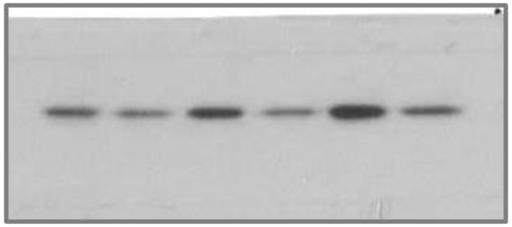

p-HH3

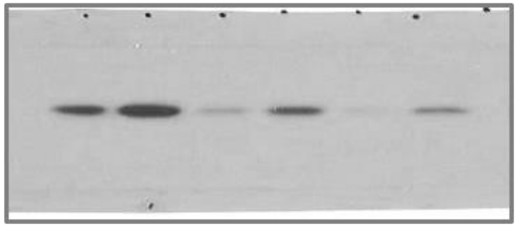

cdc2

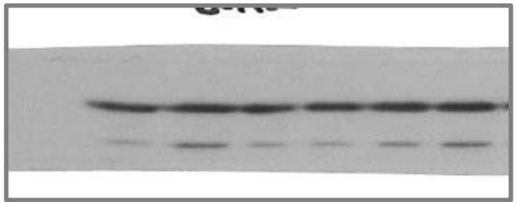

β-actin

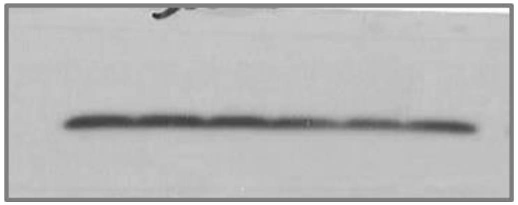

Supplement Figure 3A Unprocessed images for blots

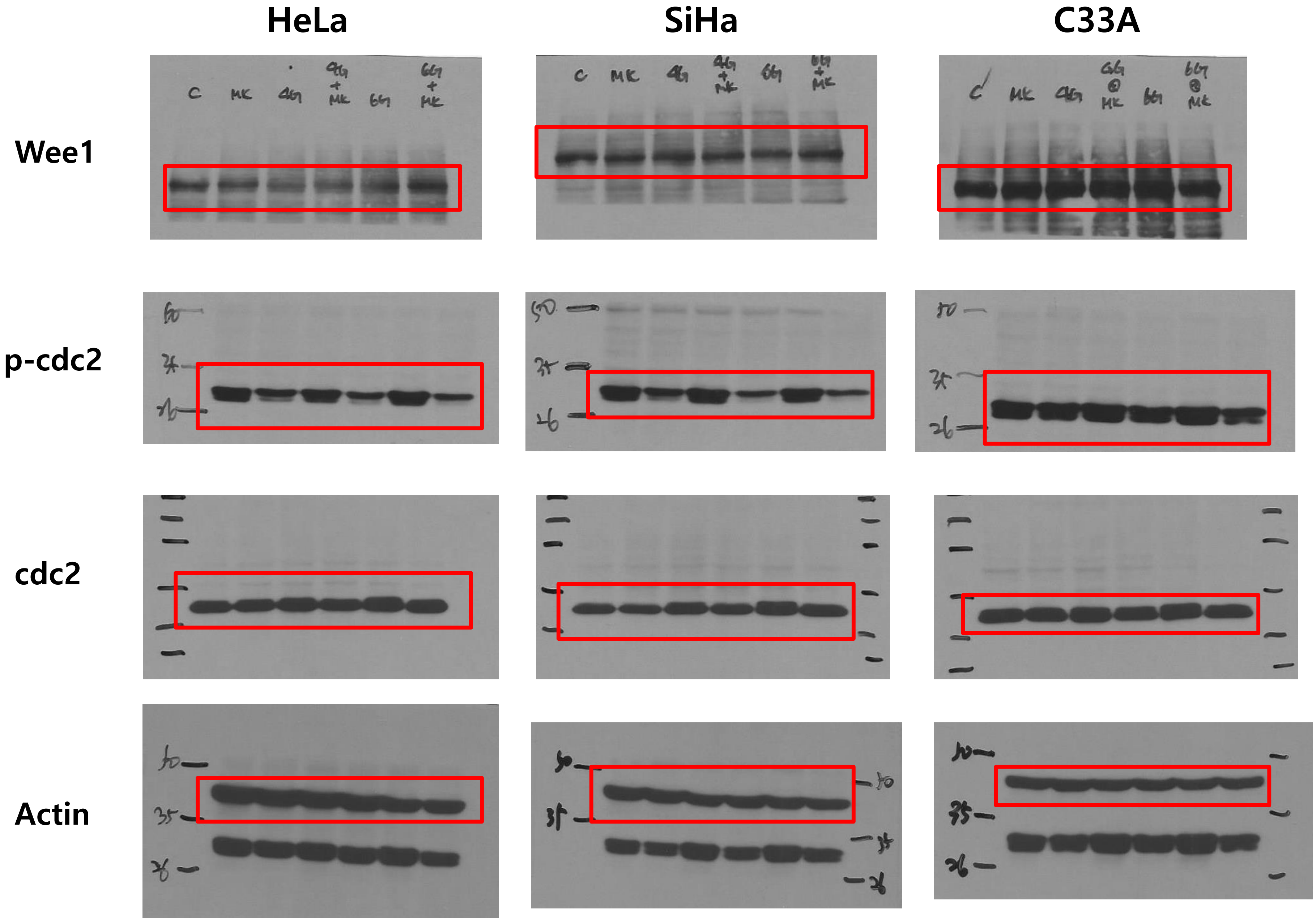

Supplement Figure 3B Unprocessed images for blots

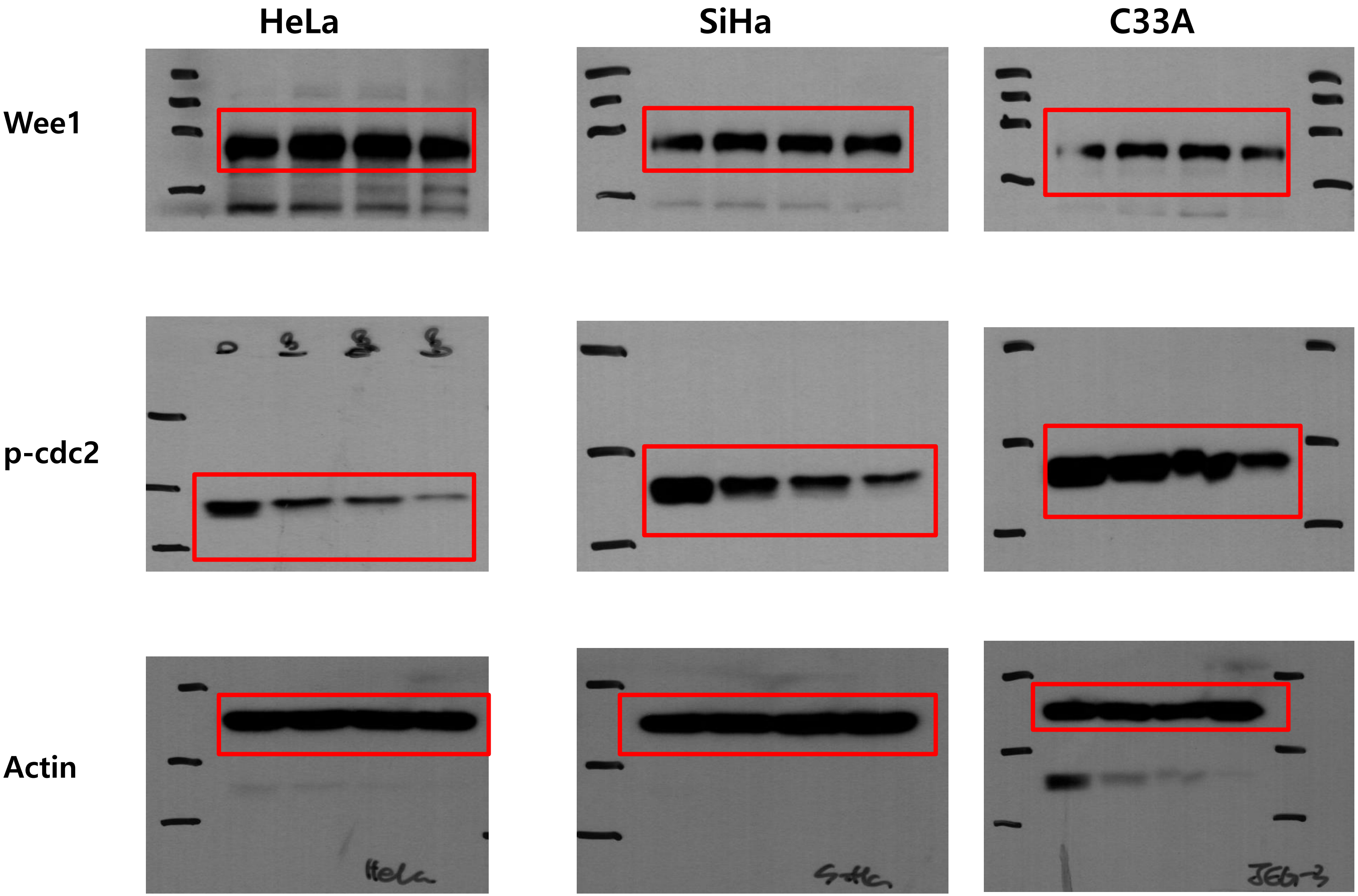

Supplement: Supplementary file 3 — Full blots [file 41598_2019_51959_MOESM3_ESM.pdf]
